# Early MRI-Derived Volumetric Thresholds Predict Response and Guide Personalization in HER2-Positive Breast Cancer: A Retrospective Study

**DOI:** 10.3390/biomedicines13122906

**Published:** 2025-11-27

**Authors:** Hao Yao, Xuyang Qian, Ran Zheng, Xingye Sheng, Jingjing Ding, Mingyu Wang, Xiaoming Zha, Shouju Wang, Jue Wang

**Affiliations:** 1 Department of Breast Disease, The First Affiliated Hospital of Nanjing Medical University, Nanjing 210029, China; yh18086791856@163.com (H.Y.); 15705227155@163.com (R.Z.); 17849860565@163.com (X.S.); 15850086268@163.com (J.D.); wmy8764@163.com (M.W.); njzhaxm@njmu.edu.cn (X.Z.); 2 Department of Radiology, The First Affiliated Hospital of Nanjing Medical University, Nanjing 210029, China; qxy15861603784@163.com

**Keywords:** breast neoplasms, receptor, ErbB-2, neoadjuvant therapy, magnetic resonance imaging

## Abstract

**Background**: Neoadjuvant systemic therapy (NST), whose primary purposes include response assessment and treatment individualization, is a key strategy in the treatment of HER2-positive breast cancer. This study investigated the predictive value of the magnetic resonance imaging (MRI)-derived tumor volume reduction rate (δV1) for the early identification of pathological complete response (pCR) during NST and established clinically applicable δV1 thresholds for patient stratification. **Methods**: HER2-positive breast cancer patients who received THP (taxane, trastuzumab, pertuzumab) followed by epirubicin/cyclophosphamide (EC) were enrolled. MRI was performed at baseline, after THP, and after EC. Tumor volumes were manually segmented using 3D Slicer, and δV1/δV2 were calculated via Python (version3.13). Longest diameter reduction rates (δL1/δL2) were recorded. pCR (ypT0/is ypN0) was the primary endpoint. Receiver operating characteristic (ROC) analysis determined predictive accuracy, and logistic regression identified independent predictors. Thresholds for δV1 were explored, and subgroup analyses were conducted by hormone receptor (HR) and human epidermal growth factor receptor 2 (HER2) status. **Results**: Overall, 59.3% of patients achieved pCR. δV1 demonstrated superior predictive accuracy compared with longest diameter reduction (δL1), with an AUC of 0.745 (95% CI: 0.642–0.847) vs. 0.634 (95% CI: 0.512–0.757). A δV1 cutoff of 0.85 discriminated responders (68.4% vs. 41.4%, *p* = 0.016), while one of 0.91 represented the optimal predictive threshold. In multivariate analysis, δV1 was independently associated with pCR (OR = 1227.1, 95% CI: 6.86–219,562; *p* = 0.007), along with HER2 3+ expression (OR = 4.24, 95% CI: 1.26–14.31; *p* = 0.020). Among HR-positive patients, δV1 < 0.93 identified a subgroup with significantly lower pCR rates (19.0% vs. 81.0%, *p* < 0.001). **Conclusions**: δV1 is a reliable and early MRI-based imaging biomarker for predicting pCR in HER2-positive breast cancer. Defining thresholds such as 0.85 and 0.91 supports early therapeutic stratification and may help identify patients who could benefit from anthracycline-containing regimens.

## 1. Introduction

According to recent data, breast cancer has become the most commonly diagnosed malignancy among women worldwide [1,2], and neoadjuvant systemic therapy (NST) has been widely adopted in its management. The use of NST, which was originally developed for patients with locally advanced or inoperable disease, has significantly expanded in recent years [3,4,5,6]. Approximately 15–20% of all breast cancers are human epidermal growth factor receptor 2 (HER2)-positive, characterized by HER2 gene amplification or protein overexpression [7]. This subtype is typically more aggressive and associated with poor prognosis. Considering that NST has been shown to improve pathological complete response (pCR) rates and prolong survival in HER2-positive patients [8,9], both domestic and international clinical guidelines recommend its use as first-line treatment for this subtype [10]. In addition to improving breast-conservation rates, the primary advantage of NST lies in its ability to evaluate in vivo therapeutic efficacy and to guide adaptive, individualized treatment strategies [5,11]. Dual HER2-targeted therapy, particularly the combination of trastuzumab and pertuzumab, has substantially increased pCR rates, subsequently becoming the standard NST regimen for HER2-positive breast cancer [12]. Hence, multiple guidelines have now recommended regimens such as TCbHP as first-line options and anthracycline-containing regimens, such as THP-EC, as second-line alternatives. However, the omission of anthracyclines remains controversial. Although recent studies suggest that anthracycline-free protocols may not be inferior, strong and consistent evidence across all patient subgroups is still lacking [13,14].

In our clinical practice, we observed that a subset of HER2-positive patients exhibited limited tumor response during the THP phase but demonstrated significant regression during the subsequent EC phase. This clinical heterogeneity suggests that anthracyclines may retain therapeutic value in certain contexts. Therefore, the early identification of patients who may benefit from EC and those who may safely avoid it is crucial. Accordingly, reliable biomarkers to support such stratification are urgently needed. Magnetic resonance imaging (MRI)-based imaging assessment offers a non-invasive and dynamic method for monitoring tumor regression during NST. In particular, early volumetric changes may better reflect biological drug sensitivity than traditional one-dimensional measurements [15,16].

The current study enrolled HER2-positive breast cancer patients with radiologically measurable primary tumors who received full-course NST. Tumor burden was assessed throughout treatment using two indicators: longest diameter reduction (δL) extracted from MRI reports and volumetric shrinkage (δV) calculated directly from source MR images. This study focused on the tumor volume reduction rate during the THP phase (δV1) as a potential early imaging biomarker for pCR prediction and aimed to determine optimal δV1 thresholds that could stratify patients according to treatment response and inform a more precise and individualized NST strategy for HER2-positive breast cancer.

## 2. Materials and Methods

### 2.1. Patients

Eligible participants included women who were newly diagnosed with operable HER2-positive breast cancer, verified by ultrasound-guided core needle biopsy. ER/PR negativity (<1% nuclear staining) and HER2 positivity [immunohistochemistry (IHC) 3+ or fluorescence in situ hybridization (FISH)-amplified] were defined based on ASCO/CAP guidelines. All patients were required to complete all cycles of taxane (T) combined with trastuzumab and pertuzumab (HP), followed by sequential epirubicin/cyclophosphamide (EC) therapy. MRI was performed before the first and fifth cycle of NST, as well as before surgery. Exclusion criteria comprised a history of chemotherapy, targeted therapy, or radiotherapy; presence of distant metastases or non-measurable lesions (ill-defined tumor boundaries or discontinuous post-NST lesions); uncontrolled systemic conditions [e.g., active infections, diabetes (HbA1c > 9%), malignant hypertension (BP ≥ 180/110 mmHg), or hemorrhagic diathesis]; organ dysfunction [e.g., LVEF < 55% (baseline echocardiography), serum creatinine > 1.5× of the upper limit of normal (ULN), AST/ALT > 1.5× ULN]; and premature NST discontinuation due to toxicity or non-compliance. Any patient with missing critical data such as tumor volume measurements at one or more timepoints was excluded from the analysis. To minimize selection bias, all eligible patients who met the inclusion criteria and completed the entire neoadjuvant treatment regimen were included in the study. Patients were enrolled consecutively, and no preferential selection was made. A total of 86 patients who met the inclusion criteria were enrolled in the study.

### 2.2. Study Design

**Figure 1:**
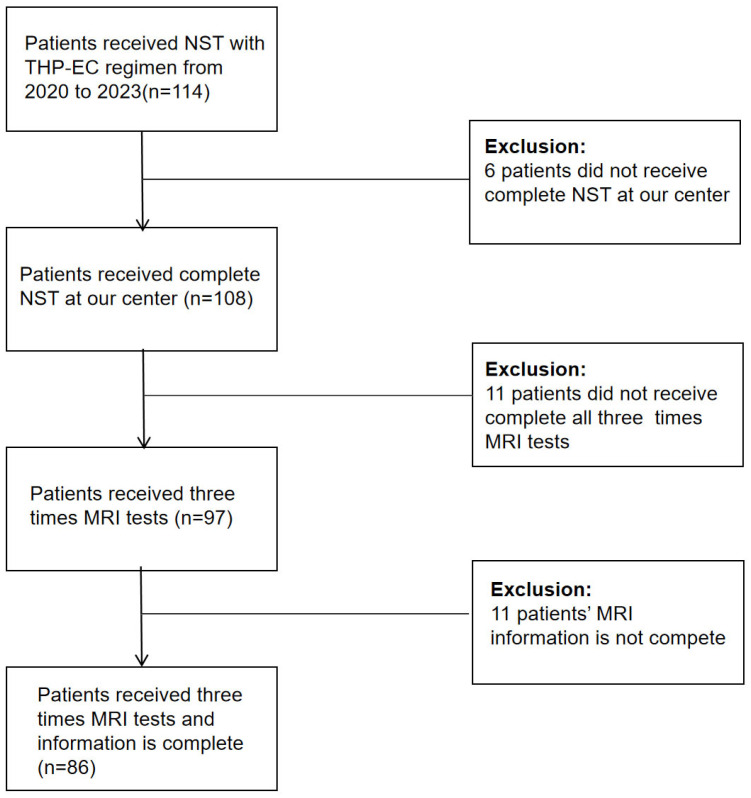
Flow diagram of patient inclusion. From an initial cohort of 114 HER2-positive breast cancer patients treated with THP-EC neoadjuvant therapy, 86 cases with complete neoadjuvant systemic therapy and three magnetic resonance imaging timepoints were retained for final analysis.

This retrospective single-center study included 86 HER2-positive invasive breast cancer patients who received the THP-EC regimen at the First Affiliated Hospital of Nanjing Medical University. All patients underwent MRI at three timepoints: before NST, after THP (taxane, trastuzumab, and pertuzumab), and after EC (Figure 1). The primary endpoint of this study was the pCR, defined as the absence of invasive carcinoma in both the breast (ypT0/is) and axillary lymph nodes (ypN0) following neoadjuvant systemic therapy (NST). This endpoint was defined a priori to evaluate the effectiveness of the treatment regimen in achieving complete tumor regression. Secondary endpoints included tumor volume reduction rate (δV1, δV2) and changes in tumor diameter (δL1, δL2) during neoadjuvant therapy, as well as the association between clinical/pathological factors (such as hormone receptor status and HER2 expression) and treatment response. These endpoints were exploratory and not predefined in the study protocol, and they should be interpreted with caution.

HER2 positivity was determined based on a score of 3+ on IHC or a score of 2+ accompanied by confirmed HER2 gene amplification via FISH. ER and PR positivity were defined as nuclear staining observed in more than 1% of tumor cells.

Prior to both NST initiation and surgical intervention, patients underwent comprehensive evaluations, including complete blood count examination, hepatic and renal function panels, and electrocardiography. Dose modifications were applied in response to adverse events. The treatment protocol began with four cycles of a taxane-based regimen combined with dual HER2 blockade. Taxane treatment was administered using various formulations: nab-paclitaxel and solvent-based paclitaxel were administered at 14-day intervals (260 and 175 mg/m^2^ on day 1, respectively), whereas docetaxel (75 mg/m^2^) was administered every 21 days. Liposomal paclitaxel (175 mg/m^2^) was also administered on day 1 of each cycle. Trastuzumab was administered with a loading dose of 8 mg/kg, followed by 6 mg/kg every 21 days, whereas pertuzumab was administered at an initial dose of 840 mg, followed by 420 mg every 21 days. This THP regimen was subsequently followed by four cycles of anthracycline-based chemotherapy consisting of epirubicin (90 mg/m^2^) and cyclophosphamide (600 mg/m^2^) administered on day 1 at intervals of either 14 or 21 days, depending on patient tolerance. Definitive surgery was scheduled approximately 4 weeks after completing neoadjuvant therapy.

MRI scans were conducted in the prone position using either a 1.5 Tesla scanner (MAGNETOM Aera XJ, Siemens). MRI was conducted at three timepoints: before NST, at the midpoint of NST, and after NST. All MRI files for a given timepoint were merged into a single file to facilitate analysis. Tumor segmentation on MRI images was performed manually using 3D Slicer (version 5.2.2) on the second post-contrast phase of DCE sequence (TR/TE, 4.23 ms/1.57 ms; matrix, 448 × 448; field of view, 340 mm × 340 mm; thickness, 1 mm). Two radiologists with over 5 years of breast imaging experience underwent standardized training before participating in the segmentation process. The training included calibration sessions using reference cases to ensure consistency in tumor boundary delineation.

**Figure 2:**
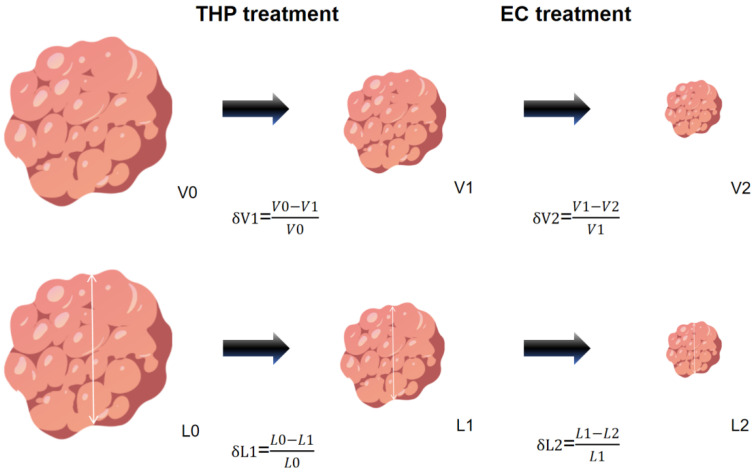
Method for calculating the volumetric change rate (δV) and long-axis diameter change rate (δL). T, taxanes; HP, trastuzumab and pertuzumab; L, long-axis diameter; δV1, volumetric change rate of THP treatment; δV2, volumetric change rate of EC phase; δL1, long-axis diameter change rate of THP phase; δL2, long-axis diameter change rate of EC treatment. Created with BioGDP.com [17].

All segmentations were performed in a blinded fashion; radiologists were unaware of patients’ clinical outcomes and each other’s assessments. For reproducibility assessment, 20 randomly selected cases were segmented independently by both readers (inter-observer agreement) and by one reader twice, one week apart (intra-observer agreement). Inter- and intra-observer reproducibility were quantified using the Intraclass Correlation Coefficient (ICC) with 95% confidence intervals. The inter-observer ICC for δV1 was 0.937 (95% CI: 0.894–0.964), and intra-observer ICC was 0.949 (95% CI: 0.916–0.972), indicating excellent agreement. Segmentation time per case ranged from 15 to 25 min. Quality control was performed through consensus review for any cases with >10% volumetric discrepancy between observers. All volumetric data were processed using a standardized Python pipeline to eliminate variability in post-segmentation analysis. Python 3.9.13 with the nibabel and numpy packages was employed to process DICOM files, extract spatial information, convert data types, and perform mathematical calculations to determine tumor volume at each timepoint. Long-axis tumor diameters were obtained from MRI reports based on the Response Evaluation Criteria in Solid Tumors (RECIST) version 1.1, which defines the maximum diameter in any orientation as the standard metric for breast cancer response monitoring. The long-axis diameter change rate during the THP phase (δL1) and EC phase (δL2) were calculated to evaluate therapeutic efficacy. Similarly, volumetric change rates during THP (δV1) and EC (δV2) phases were derived (Figure 2). pCR was defined as the absence of evidence indicating invasive disease in the breast (ypT0/is) or axillary lymph nodes (ypN0) based on histopathological examination by experienced pathologists.

### 2.3. Statistical Analysis

Differences in pCR rates between groups were assessed using Pearson’s χ^2^ test or Fisher’s exact test via IBM SPSS Statistics (version 26.0, IBM Corp, Armonk, NY, USA). The optimal threshold for δV1 was determined using the minimum absolute difference between sensitivity and specificity (min(|se − sp|)) in R (version 4.4.1, Vienna, Austria). The following packages were applied: pROC for ROC curve and AUC analysis, ggplot2 for data visualization, rms and ResourceSelection for logistic regression and calibration curves, boot for bootstrap validation, and pwr for post hoc power analysis. A two-tailed *p* value of <0.05 indicated statistical significance. Univariate logistic regression analysis was performed to identify potential predictors of pCR. Variables with a *p*-value < 0.10 in univariate analysis were included in the multivariate logistic regression model. Odds ratios (OR) and 95% confidence intervals (CI) were calculated for each variable. Model calibration was assessed using calibration curves to evaluate the agreement between predicted and observed pCR rates. ROC curve and AUC help assess the overall predictive ability of the model. Sensitivity and specificity help evaluate the model’s performance at different thresholds. Exploratory subgroup analyses were performed to investigate the relationship between pCR rates and additional biomarkers (e.g., HER2 and HR status). These analyses were not predefined and should be interpreted as hypothesis-generating. Due to the exploratory nature of these analyses, no adjustment for multiple comparisons was applied.

## 3. Results

### 3.1. Patient Characteristics

This study enrolled 86 patients with HER2-positive breast cancer who had a mean (SD) age of 50.0 (10.6) years. Most patients presented with cT2 tumors (62.7%) and axillary lymph node involvement (89.6%). HER2 3+ expression was observed in 76.7% of patients, and 59.3% achieved pCR (ypT0/is ypN0) after surgery (Table 1).

### 3.2. Volumetric Response Dynamics Between Treatment Phases

Patients with δV1 ≥ δV2 (70.9%) (Table 2) showed greater volumetric reduction during THP than during EC, whereas those with δV1 < δV2 (29.1%) showed better response during the EC phase than during the THP phase. A greater proportion of patients in the δV1 < δV2 group achieved pCR than did those in the δV1 ≥ δV2 group (64.0% vs. 57.4%), although the difference failed to reach significance.

### 3.3. Impact of Hormone Receptor (HR) Status and HER2 Expression Intensity

Patients were stratified into HR-negative (HR−, 51.2%) and HR-positive (HR+, 48.8%) subgroups (Table 2). HR− patients exhibited a higher pCR rate than did HR+ patients (68.2% vs. 50% in HR+), although the difference failed to reach significance. Among HR− patients, the prevalence of δV1 ≥ δV2 was greater than that of δV1 < δV2 (72.7% vs. 27.3%), with a similar trend having been observed among HR+ patients (70.7% vs. 29.3%). Neither subgroup demonstrated significant pCR rate differences based on δV1/δV2 dynamics. Patients with HER2 3+ had significantly higher pCR rates than did patients with HER2 2+ (68.2% vs. 30%, *p* = 0.004) (Table 2).

### 3.4. Volumetric vs. Diametric Response Metrics

Using the mean value of δV1 (0.85) as a threshold, patients with δV1 ≥ 0.85 achieved higher pCR rates than did those with δV1 < 0.85 (68.4% vs. 41.4%, *p* = 0.016) (Table 3). Subgroup analysis revealed that within the δV1 ≥ 0.85 group, those with δV1 < δV2 achieved 100.0% pCR, whereas only 62.5% in the δV1 ≥ δV2 group did the same (*p* = 0.026). In contrast, long-axis diameter change rate (δL1) thresholds (mean δL1 = 0.55) showed no significant predictive value (66.7% vs. 50%, *p* > 0.05).

### 3.5. Predictive Performance of δV1 and δL1

**Figure 3:**
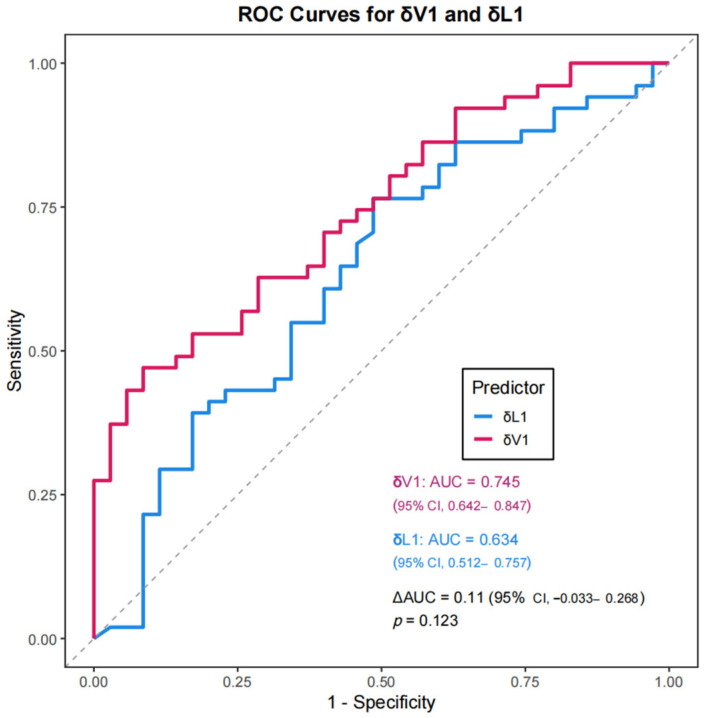
Comparison of ROC curves for δV1 and δL1 in predicting pathological complete response (pCR). The dotted line represents the level of random guessing.The AUCs were 0.745 (95% CI, 0.642–0.847) for δV1 and 0.634 (95% CI, 0.512–0.757), with a non-significant difference (ΔAUC = 0.11; 95% CI, −0.033–0.268; *p* = 0.123).

**Figure 4:**
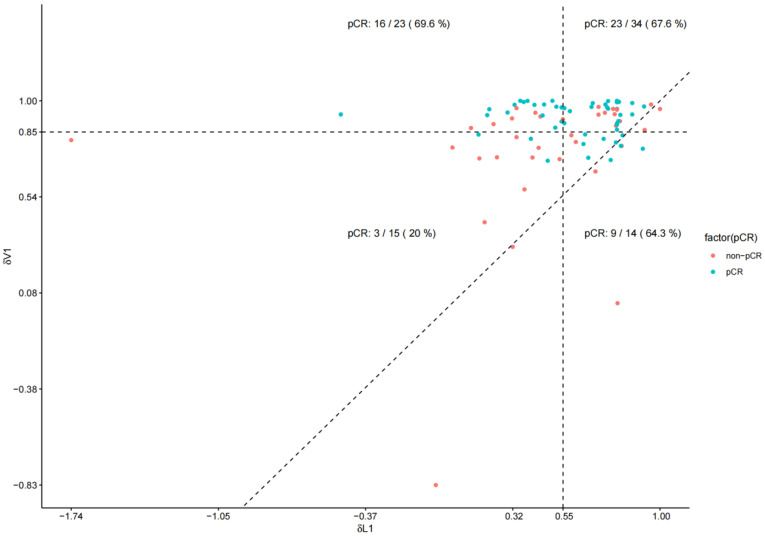
Scatter plot investigating the discrepancy in the area under the curve values between δV1 and δL1, focusing particularly on cases achieving pathological complete response despite being classified as δL1 non-responders (upper-left quadrant). The dotted lines on the x-axis and y-axis represent the mean values of δL1 and δV1, respectively, while the 45° dotted line represents the situation where the shrinkage rates of both are equal.

**Figure 5:**
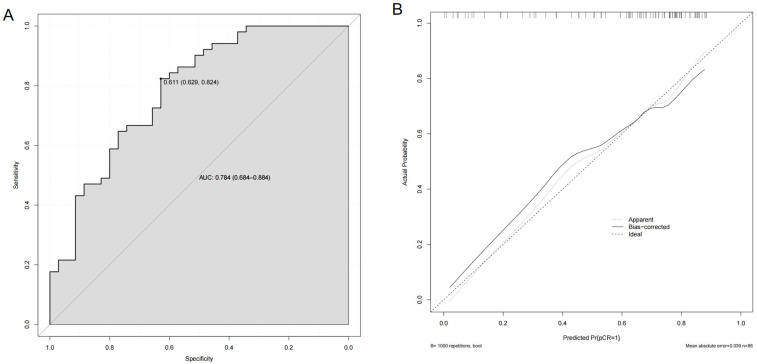
Receiver operating characteristic (ROC) and calibration curves for the multivariable logistic regression model predicting pathological complete response (pCR). (**A**) ROC curve of the multivariable model incorporating δV1, δL1, HER2 expression, HR status. The model achieved an area under the ROC curve (AUC) of 0.784 (95% CI, 0.684–0.884), indicating good discriminative ability for predicting pCR. (**B**) Calibration curve assessing agreement between predicted and observed probabilities of pCR after 1000 bootstrap resamples. The solid line represents bias-corrected estimates, the dotted line represents apparent performance, and the diagonal dashed line represents perfect calibration.

Receiver operating characteristic (ROC) analysis demonstrated superior predictive accuracy with δV1 (AUC = 0.745) (95% CI, 0.642–0.847) than with δL1 (AUC = 0.634) (95% CI, 0.512–0.757), though the difference was marginally non-significant (*p* = 0.123) (Figure 3). A scatterplot revealed that the highest and lowest pCR rates were in the upper-left quadrant (69.6%) and lower-left quadrant (20.0%), respectively (Figure 4). A multivariable logistic regression model was constructed to predict pCR (Table 4). In the multivariate analysis, both δV1 (OR = 1227.075, 95% CI: 6.858–219,561.9992, *p* = 0.007) and HER2 3+ expression (OR = 4.241, 95% CI: 1.257–14.312, *p* = 0.020) were independently associated with pCR, while δL1 was not significant (*p* = 0.605). The wide confidence interval reflects the limited sample size and the strong effect of δV1 on pCR classification, indicating that patients with higher early volumetric reduction (δV1) were markedly more likely to achieve pCR. Although the magnitude of the OR appears large, this is not uncommon when the predictor variable is continuous and standardized on a proportional scale rather than a binary variable. The result confirms that early volumetric response exerts a substantial positive association with achieving pCR. The model achieved ability with an area under the ROC curve (AUC) of 0.784 (95% CI, 0.684–0.884) (Figure 5A). Internal validation using bootstrap resampling (B = 1000) demonstrated satisfactory model calibration, with the bias-corrected curve closely approximating the ideal line and a absolute error of 0.039 (Figure 5B).

### 3.6. Optimized δV1 Threshold and Subgroup Analysis

**Figure A1:**
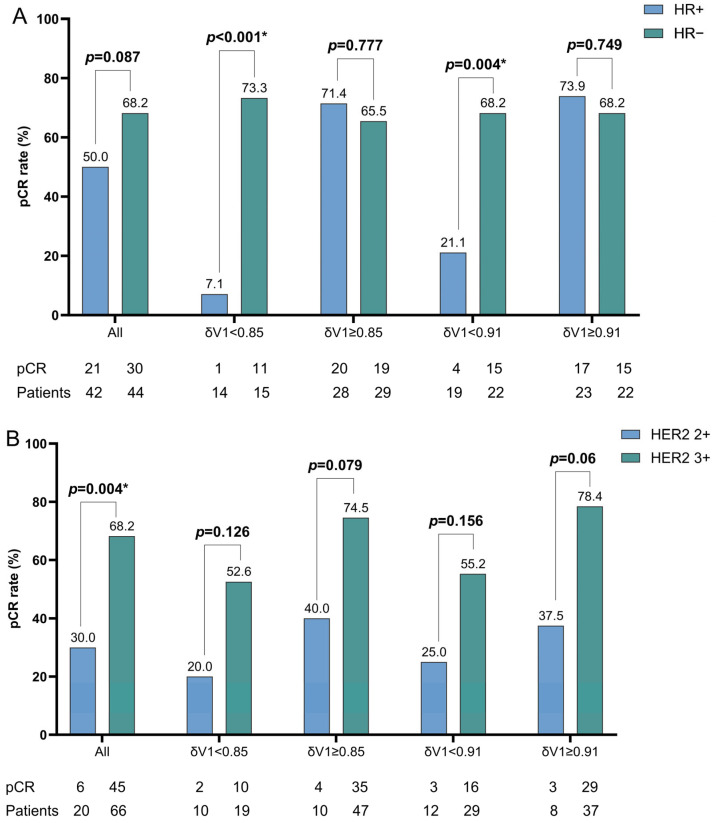
(**A**) The pCR rate of HR+ and HR− and subgroup analysis by different δV1 threshold. (**B**) The pathological complete response rate of HER2 2+ and HER2 3+ and subgroup analysis by different δV1 threshold. *, Asterisks indicate statistically significant associations (*p* < 0.05).

The analysis in Table 5 further supports the potential of δV1 as an early predictor of pCR. Using δV1 ≥ 0.91 as a threshold, patients who achieved higher early volumetric reductions demonstrated a significantly higher rate of pCR (71.1% vs. 46.3%, *p* = 0.02). This result highlights the utility of δV1 in stratifying patients based on their likelihood of achieving complete response following neoadjuvant therapy. Among HR+ patients, those with δV1 values below the subgroup-specific threshold of 0.93 showed a markedly reduced pCR rate those with δV1 values above the threshold (19.0% vs. 81.0%, *p* < 0.001) (Table A1). High HER2 expression (3+) remained consistently associated with elevated pCR rates across all subgroups (Figure A1).

## 4. Discussion

### 4.1. Research Background and Construction of Imaging-Based Evaluation Methodology

The combination of taxanes and anti-HER2 agents has served as the cornerstone of neoadjuvant chemotherapy for HER2-positive early breast cancer, consequently improving breast-conservation rates and survival outcomes [18]. Anthracyclines, which have been widely utilized in cancer therapy owing to their potent antitumor effects, not only reduce tumor volume and enhance surgical resectability but also act synergistically with large-molecule targeted therapies, such as trastuzumab or pertuzumab, to further improve efficacy [19]. The recent neoCARHP study demonstrated that in early-stage HER2-positive breast cancer patients receiving dual HER2-blockade, the THP regimen was not inferior to TCbHP in terms of pCR rate while also improving tolerability [20]. This finding suggests that omission of carboplatin may represent an effective de-escalation strategy in neoadjuvant therapy. Consequently, our institution had implemented the THP-EC sequential regimen (taxane, trastuzumab, pertuzumab followed by epirubicin/cyclophosphamide) for HER2-positive patients undergoing neoadjuvant therapy. In the current study, hormone receptor-negative (HR−) and HR+ subgroups had been nearly equally represented among the 86 patients enrolled (51.1% vs. 48.9%, respectively).

During neoadjuvant therapy, MRI was used to evaluate the relative reduction in primary tumor size before and after treatment. The observed heterogeneity in tumor volumetric change rates (relative to baseline values) reflects interpatient variability in response to the same neoadjuvant regimen, with intrapatient differences even emerging across distinct chemotherapy phases. These differences suggest variations in drug sensitivity or potential resistance to specific agents. To compare the efficacy of THP and EC, MRI data were collected at three timepoints, namely before neoadjuvant therapy, after THP, and after EC. For irregularly shaped tumors, conventional measurements, such as the longest diameter or simplified length × width × height, may inaccurately quantify volumetric reduction during chemotherapy given that they fail to capture three-dimensional tumor regression. To address this, raw MRI data were processed using 3D Slicer software (version 5.2.2) for manual segmentation of primary lesions at each timepoint. The segmented data were then imported into Python for computational analysis, which enables precise tumor volume calculations. This approach provided an objective assessment of volumetric changes across chemotherapy phases. The volumetric change rate from baseline to post-THP (δV1) was defined as the tumor volume reduction during the THP phase divided by the baseline volume. Similarly, the volumetric change rate from post-THP to post-EC (δV2) was calculated as the volume reduction during the EC phase divided by the mid-therapy volume. Concurrently, the longest diameter change rates were recorded from MRI reports, with δL1 (post-THP) and δL2 (post-EC) being derived using RECIST version 1.1. These linear metrics were compared against volumetric measurements to evaluate their concordance in therapeutic response assessment.

### 4.2. Therapeutic Differences Between THP and EC Phases and Identification of Dual-Target Resistance Subgroups

As shown in Table 2, a larger proportion of patients fell into the δL1 ≥ δL2 and δV1 ≥ δV2 categories, suggesting that most HER2-positive patients responded favorably to targeted treatment, which is consistent with clinical expectations. Despite the cardiotoxic side effects of anthracyclines, which are dose-dependent and may cause symptomatic heart failure years after treatment, thereby complicating risk assessment and prevention [21], they remain irreplaceable in certain contexts. Recent studies highlight that the PH-FECH regimen (paclitaxel/trastuzumab followed by fluorouracil, epirubicin, and cyclophosphamide) yields higher pCR rates and longer progression-free survival than does TCH (docetaxel/carboplatin/trastuzumab), underscoring the persistent role of anthracyclines in breast cancer therapy [22,23]. Although approximately 70% of patients achieved good responses with targeted therapies, 30% showed superior efficacy with EC than with THP (δV1 < δV2, Table 2). Patients with δL1 and δV1 values lower than δL2 and δV2 achieved a pCR rate of 64%, whereas those with equal or greater changes achieved a rate of 57.4% (*p* > 0.05). This trend highlights the continued importance of anthracyclines, even among patients who appear sensitive to HER2-targeted therapy, and indicates the need for caution when deciding to omit anthracyclines. Additionally, EC therapy is particularly vital for patients resistant to targeted therapies [24]. To date, no study has yet demonstrated superior pCR or disease-free survival with anthracycline-free regimens over anthracycline-containing protocols in specific populations [25], emphasizing the need for early identification of targeted therapy-resistant patients to tailor personalized regimens.

In early breast cancer, pCR rates vary according to hormone receptor (HR) status, with HR−/HER2+ patients achieving higher pCR rates than do HR+/HER2+ patients [26]. Consistent with the literature, our study observed higher pCR rates in the HR− group than in the HR+ subgroup (68.2% vs. 50%, *p* = 0.086), mirroring findings of the TRYPHAENA trial (70% vs. 50% pCR in the HR− vs. HR+ subgroups, respectively) [27]. These findings may stem from the inherently aggressive biology and heightened chemosensitivity of HR− tumors as opposed to HR+ tumors, which often exhibit poorer chemotherapy responses [28]. The absolute difference of 18.2% suggests a potentially meaningful clinical trend that warrants further investigation in a larger cohort. HER2 3+ patients achieved significantly higher pCR rates than did HER2 2+ patients (68.2% vs. 30%, *p* = 0.004), likely due to lower HER2 amplification rates in HER2 2+ tumors, which reduces target sensitivity. HER2 expression intensity has been validated as an independent predictor of pCR [26]. However, the findings from the exploratory analyses, including the potential relationship between HER2 expression and pCR, should be regarded as hypothesis-generating. These results require further validation in larger, independent cohorts and may be subject to type I error due to the lack of multiple testing correction.

To further explore the efficacy of THP in HER2-positive patients, the mean values of δL1 and δV1 in the 86 patients included herein were calculated to be 0.55 (δL) and 0.85 (δV). This represents the central tendency of the tumor volume reduction observed in this patient population. By selecting the mean value, we aimed to capture the typical magnitude of tumor shrinkage that occurs during neoadjuvant therapy. This threshold serves as a reference for classifying patients with typical or above-average responses to treatment. Our cohort was then divided into two groups using these means as thresholds (above and below the mean) for analysis. Patients with δL1 ≥ 0.55 achieved a pCR rate of 66.7%, whereas those with values below this threshold reached a pCR rate of 50% (*p* = 0.118; Table 3), although the difference failed to reach significance. After stratifying our cohort based on a δV1 threshold of 0.85, we found that patients with values ≥ 0.85 demonstrated a significantly greater likelihood of achieving pCR than did those with values below this cutoff (68.4% vs. 41.4%, *p* = 0.016; Table 3). These results preliminarily indicate that THP efficacy can predict pCR and that δV1 outperforms δL1 in distinguishing responders. Notably, patients with δV1 < 0.85 accounted for approximately one-third of the cohort, whereas those with δV1 ≥ 0.85 accounted for two-thirds of the cohort. Although the mean-based threshold failed to divide our cohort equally, the majority of our patients achieved better outcomes with THP, and the lower one-third of our cohort (e.g., δV1 = 0.2–0.3) may have included patients resistant to targeted therapies, warranting clinical attention. A subgroup analysis found that among patients with δL1 < 0.55, those classified as δL1 < δL2 achieved a pCR of 64.7%, whereas those classified as δL1 ≥ δL2 achieved a pCR of only 38.1%. However, in terms of volumetric response, both categories yielded similar pCR rates (38.5% vs. 43.6%). Among patients with δL1 ≥ 0.55, those in the δL1 < δL2 subgroup also tended to have higher pCR rates, albeit not significantly. In contrast, a significant difference in pCR rates was observed within the δV1 ≥ 0.85 cohort, with individuals with stronger EC-phase shrinkage (δV1 < δV2) achieving a 100% response, whereas those with greater THP-phase reduction achieving a 62.5% response (*p* = 0.026; Table 3), reaffirming superior discriminative power of δV1. This finding indicates that even among THP-sensitive patients, EC improves pCR in a subset of patients, necessitating caution when considering the omission of anthracyclines. Identifying biomarkers for EC sensitivity requires further investigation.

To visually compare δL1 and δV1 in predicting pCR, ROC curves were plotted (Figure 3). Accordingly, the AUC for δV1 (AUC = 0.745) (95% CI, 0.642–0.847) was higher than that for δL1 (AUC = 0.634) (95% CI, 0.512–0.757), suggesting that volumetric changes may better predict pCR,. Although this difference did not reach conventional statistical significance (*p* = 0.123), the observed effect size suggests a potentially meaningful advantage, which should be interpreted with caution given the uncertainty. A scatterplot comparing δL1 and δV1 with a 45° reference line (Figure 4) revealed that most patients fell above this line, indicating greater volumetric reduction than diameter reduction. This “volume-first shrinkage” pattern likely reflects rapid tumor necrosis or density reduction, directly correlating with higher pCR rates. Notably, 61% (23/38) of the patients classified as δL1 non-responders were reclassified as responders by δV1, with 69.6% (16/23) achieving pCR. This discrepancy suggests that δL1 alone was inadequate for assessing response in cases with substantial volumetric but minimal diameter changes, explaining the divergent AUC values. Although some patients in the lower right quadrant were still misclassified by δV1, its false-negative rate was lower than that of δL1 (23.5% vs. 37.3%). Our multivariable model combining δV1 and clinicopathological features demonstrated robust discriminative performance (AUC = 0.784, 95% CI: 0.684–0.884) and excellent calibration after bootstrap internal validation. These findings support δV1 as a strong early imaging biomarker for pCR prediction. Although δL1 also showed moderate performance, it did not remain significant in multivariate analysis, highlighting the added value of volumetric over diametric measurements. Importantly, the model maintained reliable calibration with minimal overfitting (mean absolute error = 0.039), indicating that its predictions were well-aligned with actual pCR rates. However, given the relatively small sample size and retrospective design, further external validation in larger cohorts is warranted. The post hoc power analysis indicated that our study had an exceptionally high statistical power of 99.83% (*p* = 0.05). This suggests that the study was well-powered to detect significant effects, and the absence of significant differences between groups in the current analysis can be attributed to the true absence of a clinically meaningful difference, rather than a lack of statistical power. Overall, our findings suggest that δV1 more comprehensively reflects tumor shrinkage during therapy than does δL1.

### 4.3. Determination of δV1 Predictive Thresholds and Subgroup Stratification Performance

Based on previous results, patients with δV1 below the mean had significantly lower pCR rates than did those with δV1 above the mean, demonstrating that δV1 can quantitatively predict THP efficacy. In the δV1 ≥ mean subgroup, 84.2% of patients exhibited δV1 ≥ δV2, whereas only 15.8% showed δV1 < δV2 (Table 3), indicating a marked imbalance in subgroup distribution. This finding suggests that the majority of chemotherapy-sensitive patients derive their benefits primarily from THP. Notably, the δV1 < δV2 subgroup achieved a 100% pCR rate, highlighting that even among patients with favorable THP responses, a small subset can achieve superior pCR through EC, underscoring its indispensable role. The threshold of 0.91 was determined using the minimum absolute difference between sensitivity and specificity (min|se − sp|), a commonly used approach to optimize diagnostic thresholds. This method aims to identify the point at which both sensitivity and specificity are balanced, providing the best overall discrimination between responders and non-responders. By choosing this threshold, we sought to maximize the predictive accuracy of δV1 in identifying patients who would benefit from neoadjuvant therapy. The cohort was nearly evenly divided into δV1 ≥ 0.91 (52.3%) and δV1 < 0.91 (47.7%) subgroups (Table 4). The δV1 ≥ 0.91 subgroup demonstrated higher pCR rates compared with those with δV1 < 0.91 (71.1% vs. 46.3%, *p* = 0.02). While this threshold showed potential discriminative value, these findings are exploratory and should be validated in independent cohorts before clinical application. Among patients with δV1 ≥ 0.91, a majority (80%) demonstrated attenuated response during subsequent EC treatment, which was associated with a reduced pCR rate of 63.9% (*p* = 0.042; Table 4). This finding suggests that prolonging dual-targeted therapy may benefit this subgroup. Both of these thresholds (0.85 and 0.91) were derived from the same cohort of patients, making them exploratory findings. As such, these thresholds require further external validation in independent cohorts before they can be applied in clinical practice.

To investigate whether the δV1 threshold could accurately stratify HER2-positive breast cancer patients with differing pCR rates across hormone receptor status and HER2 expression intensity, a bar chart was generated for comparison (Figure A1). Regarding hormone receptor status, the HR− group exhibited a higher pCR rate than did the HR+ group in the overall population, as previously described. Subgroup analyses using δV1 thresholds of 0.85 and 0.91 revealed that the lower pCR rate in HR+ patients stemmed from exceptionally poor outcomes in δV1-defined non-responders (HR+). However, among δV1-defined responders, HR− and HR+ patients had comparable pCR rates. When calculating δV1 means separately for HR+ and HR− cohorts, we found that HR+ patients with δV1 below their subgroup-specific mean (0.93) showed significantly lower pCR rates than did those with δV1 above the mean (19.0% vs. 81.0%, *p* < 0.001; Table A1). This finding indicates that HR+ patients with limited early tumor regression (δV1 < subgroup mean) require heightened clinical scrutiny, with the necessity of treatment escalation warranting further study. Despite the high level of significance, these findings are based on subgroup analysis and should be interpreted cautiously. The large effect size highlights the potential utility of δV1 for stratification, pending further validation. Similarly, a comparison of the HER2 2+ and HER2 3+ subgroups showed significant pCR differences in the overall population. After δV1 stratification, the pCR rate gap between HER2 2+ and HER2 3+ patients approached significance in the δV1-defined responder subgroups. HER2 3+ patients consistently achieved high pCR rates across all subgroups, whereas HER2 2+ (low HER2 expression) was associated with inferior therapeutic responses, confirming HER2 expression intensity as a critical predictor of treatment efficacy.

The volumetric reduction rate during early treatment (δV1) may serve as a non-invasive imaging biomarker to identify patients who are likely to achieve pCR early during neoadjuvant therapy. Clinically, this metric could be used after completion of the THP (trastuzumab + pertuzumab) phase to stratify patients into responders vs. non-responders. Those with insufficient volumetric response (e.g., δV1 < 0.85) may be considered for treatment escalation, closer monitoring, or enrollment into clinical trials, while strong responders could potentially benefit from therapy de-escalation or earlier surgery. Nevertheless, relying solely on δV1 for decision-making carries risks of misclassification. False positives may lead to overtreatment or premature surgery, whereas false negatives could delay necessary escalation of therapy. Therefore, δV1 should be integrated with other clinical and pathological features in a multimodal decision framework until prospective validation is achieved.

### 4.4. Study Limitations and Future Perspectives

This study has several limitations. First, it was conducted at a single center with a retrospective design, which may introduce selection bias and limit the generalizability of the findings. Second, the modest sample size (*n* = 86) may affect statistical power, particularly in subgroup analyses, and increases the risk of overfitting in multivariable models. Third, spectrum bias may have occurred due to the inclusion of a specific subtype of HER2-positive breast cancer patients receiving a uniform neoadjuvant regimen, which may not reflect the broader patient population. Fourth, the predictive model was developed and internally validated within the same cohort, lacking external validation in independent datasets. This limits the current applicability of the δV1 threshold in diverse clinical settings. Lastly, segmentation of MRI-derived tumor volumes involved semi-manual processes, which are inherently subject to intra- and inter-observer variability, despite the use of standardized protocols and experienced readers. Given the single-center, retrospective nature of this study and the relatively modest sample size, future prospective validation is required to confirm the predictive utility of δV1. An appropriately powered multi-center trial should be designed to include patients with HER2-positive breast cancer undergoing standardized neoadjuvant regimens. Based on an estimated effect size from our cohort (AUC ~0.75), a sample size of approximately 250–300 patients may be needed to ensure sufficient statistical power. Imaging protocols, segmentation workflow, and response criteria should be harmonized across sites to allow reproducible implementation in clinical workflows.

## 5. Conclusions

This study demonstrates that the MRI-derived volumetric reduction rate after initial THP therapy (δV1) is a reliable and early predictor of pathological complete response in HER2-positive breast cancer. Compared with conventional one-dimensional RECIST measurements, δV1 provides a more accurate and dynamic reflection of tumor biology during NST. A δV1 threshold of 0.85 and 0.91 effectively distinguishes responders from non-responders and identifies patients who may benefit from continued anthracycline-based therapy. In HR-positive patients, limited volumetric reduction (δV1 < 0.93) is associated with substantially lower pCR rates, indicating a need for treatment intensification or regimen adjustment. Integration of δV1 into early MRI evaluations offers a practical approach to guide personalized neoadjuvant treatment, potentially reducing overtreatment and improving precision in HER2-positive breast cancer management. Prospective multicenter validation is warranted to confirm these findings and facilitate clinical implementation.

## Figures and Tables

**Table 1:** Baseline clinical and pathological features of enrolled human epidermal growth factor receptor 2 (HER2)-positive breast cancer patients.

Characteristics	No. of Patients	Percentage
Age (years)	50.0 (10.6) ^a^	
Menstrual status		
Premenopausal	32	37.2
Postmenopausal	54	62.8
cT at diagnosis		
cT1	3	3.4
cT2	54	62.7
cT3	22	25.5
cT4	7	8.1
cN at diagnosis		
cN0	9	10.4
cN1	25	29.0
cN2	30	34.8
cN3	22	25.5
HR status		
HR−	44	51.2
HR+	42	49.8
Ki67 expression		
≥20	76	88.4
<20	10	11.6
HER2 expression		
HER2 2+	20	23.3
HER2 3+	66	76.7
Pathological Response (Post-NST)		
pCR (ypT0/is, ypN0)	51	59.3
non pCR	35	40.7

cT, clinical tumor stage; cN, clinical nodal stage; NST, neoadjuvant systemic therapy; pCR, pathological complete response (defined as absence of invasive carcinoma in both breast and axillary lymph nodes, i.e., ypT0/is ypN0); HER2, human epidermal growth factor receptor 2; HR, hormone receptor, ^a^, values are reported as mean (SD).

**Table 2:** Comparisons of pathological complete response (pCR) rates by δL and δV metrics across treatment phases and patient subgroups.

Subgroup	δV and δL	No. of Patients (%)	pCR (*n*, %)	*p*-Value
Patients (*n* = 86)	δV1 ≥ δV2	61 (70.9)	35 (57.4)	0.570
	δV1 < δV2	25 (29.1)	16 (64.0)	
	δL1 ≥ δL2	59 (68.6)	32 (54.2)	0.158
	δL1 < δL2	27 (31.4)	19 (70.0)	
HR− (*n* = 44)		44 (51.2)	30 (68.2)	0.087
	δV1 ≥ δV2	32 (72.7)	21 (65.6)	0.817
	δV1 < δV2	12 (27.3)	9 (75.0)	
	δL1 ≥ δL2	34 (77.3)	21 (61.6)	0.194
	δL1 < δL2	10 (22.7)	9 (90.0)	
HR+ (*n* = 42)		42 (48.8)	21 (50.0)	
	δV1 ≥ δV2	29 (69.0)	14 (48.3)	0.739
	δV1 < δV2	13 (31.0)	7 (53.8)	
	δL1 ≥ δL2	25 (59.5)	11 (44.0)	0.346
	δL1 < δL2	17 (40.5)	10 (58.8)	
HER2 2+ (*n* = 20)		20 (23.3)	6 (30.0)	0.004 *_a_
	δV1 ≥ δV2	13 (65.0)	3 (23.1)	0.613
	δV1 < δV2	7 (35.0)	3 (42.9)	
	δL1 ≥ δL2	13 (65.0)	3 (23.1)	0.613
	δL1 < δL2	7 (35.0)	3 (42.9)	
HER2 3+ (*n* = 66)		66 (76.7)	45 (68.2)	
	δV1 ≥ δV2	48 (72.7)	32 (66.7)	0.666
	δV1 < δV2	18 (27.3)	13 (72.2)	
	δL1 ≥ δL2	46 (69.7)	29 (63.0)	0.174
	δL1 < δL2	20 (30.3)	16 (80.0)	

pCR, pathological complete response; HR, hormone receptor; δL1, change in tumor longest diameter rate during the THP phase; δL2, change in tumor longest diameter rate during the EC phase; δV1, volumetric change rate during the THP phase; δV2, volumetric change rate during the EC phase; _a_, comparison of pCR rates between HER2 2+ and HER2 3+, *, Asterisks indicate statistically significant associations (*p* < 0.05).

**Table 3:** pathological complete response (pCR) rates according to mean-based δV1 and δL1 thresholds and phase-specific shrinkage patterns.

	δv				δL		
Classification Criteria	Ptients (*n*, %)	pCR (*n*, %)	*p*-Value	Classification Criteria	Ptients (*n*, %)	pCR (*n*, %)	*p*-Value
All patients	86 (100.0)	51 (59.3)		All patients	86 (100.0)	51 (59.3)	
δV1 < 0.85	29 (33.7)	12 (41.4)	0.016 *_a_	δL1 < 0.55	38 (44.2)	19 (50)	0.118
δv1 ≥ δv2 subgroup	13 (60)	5 (38.5)	1.000	δL1 ≥ δL2 subgroup	21 (55.3)	8 (38.1)	0.191
δv1 < δv2 subgroup	16 (40)	7 (43.6)		δ L1 < δL2 subgroup	17 (44.7)	11 (64.7)	
δV1 ≥ 0.85	57 (66.3)	39 (68.4)		δL1 ≥ 0.55	48 (55.8)	32 (66.7)	
δV1 ≥ δV2 subgroup	48 (81.4)	30 (62.5)	0.026 *_b_	δL1 ≥ δL2 subgroup	38 (79.2)	24 (66.7)	0.315
δV1 < δV2 subgroup	9 (18.6)	9 (100)		δL1 < δL2 subgroup	10 (20.8)	8 (80)	

pCR, pathological complete response; δL1, change in tumor longest diameter rate during the THP phase; δL2, change in tumor longest diameter rate during the EC phase; δV1, volumetric change rate during the THP phase; δV2, volumetric change rate during the EC phase. 0.85 represents the mean δV1 value; 0.55 indicates the average δL1 level; _a_, comparison of pCR rates between δV1 ≥ 0.85 and δV1 < 0.85 group; _b_, comparison of pCR rates between EC-dominant (δV1 < δV2) and THP-dominant (δV1 ≥ δV2), *, Asterisks indicate statistically significant associations (*p* < 0.05).

**Table 4:** Univariate and multivariate logistic regression analyses for predictors of pathological complete response (pCR) in patients with human epidermal growth factor receptor 2 (HER2)-positive breast cancer.

Variable	Total (*n*)	Univariate Analysis		Multivariate Analysis	
		Odds Ratio (95% CI)	*p* Value	Odds Ratio (95% CI)	*p* Value
δV1	86	1859.960 (14.163–244,263.326)	0.002	1227.075 (6.858–219,561.999)	0.007 *
δL1	86	4.135 (0.923–18.511)	0.063	1.555 (0.292–8.285)	0.605
HR status	86				
Negative	44	Reference		Reference	
Positive	42	0.467 (0.194–1.121)	0.088	0.529 (0.182–1.540)	0.243
HER2 Expression	86				
2+	20	Reference		Reference	
3+	66	5.000 (1.685–14.836)	0.004 *	4.241 (1.257–14.312)	0.020 *
Ki67	86				
≤20%	10	Reference			
>20%	76	0.326 (0.065–1.637)	0.173		
cT	86				
1	3	Reference			
2	54	3.400 (0.290–39.923)	0.330		
3	22	2.000 (0.157–25.404)	0.593		
4	7	5.000 (0.273–91.518)	0.278		
cN	86				
0	8	Reference			
1	25	0.361 (0.061–2.146)	0.263		
2	30	0.500 (0.086–2.904)	0.440		
3	23	0.519 (0.085–3.156)	0.476		

δL1, change in tumor longest diameter rate during the THP phase; δV1, volumetric change rate during the THP phase; HR, hormone receptor; HER2, human epidermal growth factor receptor 2; cT, clinical tumor stage; cN, clinical nodal stage. *, Asterisks indicate statistically significant associations (*p* < 0.05).

**Table 5:** δV1 = 0.91 served as an approximate median split for the cohort.

Classification Criteria	Patients (*n*, %)	pCR (*n*, %)	*p*-Value
δV1 < 0.91	41 (47.7)	19 (46.34)	0.020 *_a_
δV1 ≥ δV2 subgroup	25 (61.0)	12 (48.0)	1.000
δV1 < δV2 subgroup	16 (39.0)	7 (43.8)	
δV1 ≥ 0.91	45 (52.3)	32 (71.1)	
δV1 ≥ δV2 subgroup	36 (80.0)	23 (63.9)	0.042 *_b_
δV1 < δV2 subgroup	9 (20.0)	9 (100.0)	

δV1, volumetric change rate during the THP phase; δV2, volumetric change rate during the EC phase. 0.91 indicates the selected threshold for δV1; _a_, comparison of pCR rates with the δV1 ≥ 0.91 subgroup; _b_, comparison of pCR rates with the δV1 < δV2 subgroup. *, Asterisks indicate statistically significant associations (*p* < 0.05).

## Data Availability

The datasets analyzed during the current study are not publicly available due to ethical restrictions, but can be made available upon reasonable request from qualified researchers. Interested parties can contact the corresponding author for access to the data. The analysis code is available upon request. Interested researchers can contact the corresponding author to obtain the scripts for reproducibility of the analysis pipeline.

## Abbreviations

The following abbreviations are used in this manuscript:

NST	Neoadjuvant systemic therapy
MRI	magnetic resonance imaging
RECIST	Response Evaluation Criteria in Solid Tumors
δL	long-axis diameter change rate during the THP phase (δL1) and EC phase (δL2)
δV	volumetric change rates during THP (δV1) and EC (δV2) phases
pCR	pathological complete response
THP	taxane, trastuzumab, pertuzumab
EC	epirubicin/cyclophosphamide
HR	hormone receptor
HER2	human epidermal growth factor receptor 2
IHC	immunohistochemistry
FISH	fluorescence in situ hybridization
ER	estrogen receptor
PR	progesterone receptor

## Appendix A

**Table A1:** Comparisons of pCR rates between HR+ and HR- Patients.

Classification Criteria	Patients (%)	pCR (%)	*p*-Value	Classification Criteria	Paients (%)	pCR (%)	*p*-Value
HR+ patients	42 (100)	21 (50.0)		HR− patients	44 (100)	30 (68.2)	
δV1 < 0.93	21 (50.0)	4 (19.0)	<0.001 *_a_	δV1 < 0.91	22 (50.0)	15 (68.2)	1.000
δV1 ≥ δV2 subgroup	14 (66.7)	3 (21.4)	1.000	δV1 ≥ δV2 subgroup	13 (59.1)	9 (69.2)	1.000
δV1 < δV2 subgroup	7 (33.3)	1 (14.3)		δV1 < δV2 subgroup	9 (40.9)	6 (66.7)	
δV1 ≥ 0.93	21 (50.0)	17 (81.0)		δV1 ≥ 0.91	22 (50.0)	15 (68.2)	
δV1 ≥ δV2 subgroup	15 (71.4)	11 (73.3)	0.281	δ V1 ≥ δV2 subgroup	19 (86.4)	12 (63.0)	0.523
δV1 < δV2 subgroup	6 (28.6)	6 (100.0)		δV1 < δV2 subgroup	3 (15.7)	3 (100.0)	

0.93 and 0.91 represent the subgroup-specific δV1 threshold for HR-positive and HR-negative groups, respectively; ᵃ, Indicates comparison of pCR rates with the δV1 ≥ 0.93 group; *, Asterisks indicate statistically significant associations (*p* < 0.05).
